# Supplementary material for: A survey of patient and public perceptions and awareness of SARS-CoV-2-related risks among participants in India and South Africa
Source: PLOS Glob Public Health. 2023 Jul 10;3(7):e0001078. doi: 10.1371/journal.pgph.0001078 (PMC10332587; doi:10.1371/journal.pgph.0001078)
Supplement: Attachment — Submitted filename: Responses to Editor _ 28 Dec 2022.pdf [file pgph.0001078.s006.pdf]

Responses to Editor’s Comments

Editor’s Comments	Authors’ Responses
Thank you for submitting your manuscript entitled "A survey of patient and public perceptions and awareness of SARS-CoV-2-related risks among participants in India and South Africa" to PLOS Global Public Health. Your manuscript files have been checked in-house, but before we can proceed we need you to address the following issues:	
1. Please ensure that Funding Information and Financial Disclosure Statement are matched.	We have revised the information on **Funding sources** in the submitted manuscript to match with that provided in **Funding Information** in the online submission.
2. Please ensure that the Title in your manuscript file and the Title provided in your online submission form are the same.	The Title in the manuscript file has been revised to match that in the online submission form.
