# Supplementary material for: A survey of patient and public perceptions and awareness of SARS-CoV-2-related risks among participants in India and South Africa
Source: PLOS Glob Public Health. 2023 Jul 10;3(7):e0001078. doi: 10.1371/journal.pgph.0001078 (PMC10332587; doi:10.1371/journal.pgph.0001078)
Supplement: Attachment — Submitted filename: PGPH-D-22-01370R1 - Edits requested on submission.docx [file pgph.0001078.s005.docx]

**ISSUES TO BE ADDRESSED**

The authors wish to thank the PLOS Global Public Health team for highlighting these additional revisions that are required.

We have responded to the issues raised – please see in the table below.

| **Comments** | **Authors’ Reponses** |
| --- | --- |
| 1. Please ensure that the Title in your manuscript and the Title in your online submission form are the same. | We have revised the titles in the manuscript to the same as we have in the online submission form.  While revising the submitted manuscript, we noted that the paper may not be mainly about risks but could also reflect infection prevention measures in general. In line with this, may we revise the title **FROM**:  *A survey of patient and public perceptions and awareness of SARS-CoV-2-related risks among participants in India and South Africa*  **TO**  *A survey of patient and public perceptions and awareness of SARS-CoV-2-related risks and infection prevention measures in India and South Africa?*  We thank you for your kind consideration of this request. |
| 2. Please upload a copy of Figure 2 which you refer to in your text on pages 16 and 17. Or if the figure is no longer to be included as part of the submission please remove all reference to it within the text.  For more information, please see our guidelines: [https://journals.plos.org/globalpublichealth/s/figures](https://protect-za.mimecast.com/s/-6JbC1jp84SMDVV2ECYkndh) | We apologise for this oversight and have uploaded a copy of Figure 2. |
| 3. We have amended your Competing Interest statement to comply with journal style. We kindly ask that you double check the statement and let us know if anything is incorrect: "The authors have declared that no competing interests exist." | The statement is correct – we have revised this in the manuscripts; the clean version and the version with tracked changes. Thank you for the opportunity to check. |
| 4. We note that your file, "PGPH-D-22-01370_Submission to ECCMID 2021.pdf" contains potentially copyrighted logos. Please note that PLOS is unable to publish logos of any kind. At this time, we kindly ask the authors to remove the logos and submit a clean version of this file. | We have removed the logos and resubmitted a clean version of this file. |
