# Supplementary material for: A survey of patient and public perceptions and awareness of SARS-CoV-2-related risks among participants in India and South Africa
Source: PLOS Glob Public Health. 2023 Jul 10;3(7):e0001078. doi: 10.1371/journal.pgph.0001078 (PMC10332587; doi:10.1371/journal.pgph.0001078)
Supplement: Attachment — Submitted filename: PGPH-D-22-01370_Responses to Reviewers Comments_III.docx [file pgph.0001078.s007.docx]

**JOURNAL REQUIREMENTS**

| **Comments** | **Authors’ Reponses** |
| --- | --- |
| Please review your reference list to ensure that it is complete and correct. If you have cited papers that have been retracted, please include the rationale for doing so in the manuscript text, or remove these references and replace them with relevant current references. Any changes to the reference list should be mentioned in the rebuttal letter that accompanies your revised manuscript. If you need to cite a retracted article, indicate the article’s retracted status in the References list and also include a citation and full reference for the retraction notice. | We have reviewed our reference list and confirm that the references are complete and correct. We have not cited papers that have been retracted. We have however included additional references in our revised submission. In line with requests by the reviewers following our earlier submission, we revised various sections of the manuscript and included more information and their corresponding references – in the Introduction, Methods, and Discussion sections. This resulted in the insertion of reference numbers in these sections (Introduction, Methods, and Discussion sections) which affected the numbering of references related to subsequent sections. We have highlighted these additional and revised references in the References section of the submitted manuscript with tracked changes, labelled **PGPH-D-22-01370_Manuscript with Track Changes_v5**. |

**Comments from Reviewers**

**Reviewer #1**

| **Reviewer’s comments to Author** | **Authors’ responses** |
| --- | --- |
| **6. Review Comments to the Author**  Please use the space provided to explain your answers to the questions above. You may also include additional comments for the author, including concerns about dual publication, research ethics, or publication ethics. (Please upload your review as an attachment if it exceeds 20,000 characters)  **Reviewer #1:** The revised paper is much improved. The authors have addressed my comments but there is one important comment that they explain in their response has not been made in the paper itself. The major revision is for the authors to add their (adapted)response to my comment belowin the paper itself I encourage the authors to add pertinent details to the paper itself for my comment related to the following 'The current sampling shows a major skew of respondents towards India needs to be discussed as it has affected the results, moreover, it seems data was only collected from a specific region or state of India and not whole of India. The authors are advised to correct details wherever applicable to specify that the study is only from a select population' | The authors thank the reviewer for pointing this out. We have further revised the results (Table 1) to better reflect the sections of both countries where most of the respondents reside in, to highlight that the results are not necessarily representative of the public population of both countries. In addition, we have included this shortcoming in the Discussion section of the manuscript (paragraph 2 of Discussion):  *The sampling shows a major skew of respondents towards India. We believe that use of the paper version of the form in India (which could not be replicated for South Africa at the time of data collection) allowed the research team in India to personally engage with potential participants which may have influenced and increased the participant pool in India (Table 1). While the intention was to collect data with increased representation across both countries, this was not readily achieved. The survey invitation was distributed through the network of the researchers who are largely healthcare workers – some of whom were involved in the pandemic response. Majority of the respondents to this survey of patients and the public were resident in the province or state where the research team worked (Table 1); as such, the results discussed here are not necessarily representative of views from residents of the two countries.* |

**Reviewer #4**

| **Reviewer’s comments to Author** | **Authors’ responses** |
| --- | --- |
| All my comments in the last review have been properly addressed. However, The authors should please clarify: In line 56/57, you mentioned that your main outcome measures were awareness of SARS-CoV-2-related risks and perception of infection risks. However, between lines 261-265, in your logistic regression analysis, your response variable which should be your outcome or dependent variable was Covid-19 vaccination (‘if people were willing to take the vaccine'=1, if not=0). Can you please explain this discrepancy? Was Covid-19 vaccine uptake used as proxy for awareness level? If not, how was awareness measured or categorized for logistic regression? e.g high level or low level, aware or unaware? | The authors thank the reviewer for the point raised; please see response below:  *When looking at perceptions and awareness, some questions in the survey were related to this. There may not have been a simply way to amalgamate them to categorise participants in levels of either.*  *We also considered what information was deemed vital as people may know, for example, how to prevent the spread of COVID-19 but not what to do when someone already has COVID-19. Which of these is more important, or are they equally vital?*  *Our assumption was that if people were willing to take the vaccine, then they were aware of why the vaccine was necessary as they perceived it as something positive whereas if someone was unwilling to take the vaccine, they were assumed not to be aware of its importance and therefore have a low level of COVID-19 perception and awareness.*  *Of course, with this assumption, certain groups of people will be misclassified; an example could be those who are COVID-19 aware but refuse the vaccine for religious reasons. On the other hand, there may also be those that have no knowledge of COVID-19 but are willing to get a vaccine only because they understand the need for a vaccine (for work or other reasons) and not necessarily because they understand the dangers of COVID-19. There isn’t always a fine line between perception and awareness and so the binary question was used to encapsulate the topics and produce a workable statistic.*  We have included the above in the **Abstract** and under **Strengths and Limitations** of the study. |
